# Pursuer Assignment and Control Strategies in Multi-Agent Pursuit-Evasion Under Uncertainties

**DOI:** 10.3389/frobt.2021.691637

**Published:** 2021-08-17

**Authors:** Leiming Zhang, Amanda Prorok, Subhrajit Bhattacharya

**Affiliations:** ^1^ Department of Mechanical Engineering and Mechanics, Lehigh University, Bethlehem, PA, United States; ^2^ Department of Computer Science and Technology, Cambridge University, Cambridge, United Kingdom

**Keywords:** multi-robot systems, pursuit-evasion, probabilistic robotics, redundant robots, assignment

## Abstract

We consider a pursuit-evasion problem with a heterogeneous team of multiple pursuers and multiple evaders. Although both the pursuers and the evaders are aware of each others’ control and assignment strategies, they do not have exact information about the other type of agents’ location or action. Using only noisy on-board sensors the pursuers (or evaders) make probabilistic estimation of positions of the evaders (or pursuers). Each type of agent use Markov localization to update the probability distribution of the other type. A search-based control strategy is developed for the pursuers that intrinsically takes the probability distribution of the evaders into account. Pursuers are assigned using an assignment algorithm that takes redundancy (i.e., an excess in the number of pursuers than the number of evaders) into account, such that the total or maximum estimated time to capture the evaders is minimized. In this respect we assume the pursuers to have clear advantage over the evaders. However, the objective of this work is to use assignment strategies that minimize the capture time. This assignment strategy is based on a modified Hungarian algorithm as well as a novel algorithm for determining assignment of redundant pursuers. The evaders, in order to effectively avoid the pursuers, predict the assignment based on their probabilistic knowledge of the pursuers and use a control strategy to actively move away from those pursues. Our experimental evaluation shows that the redundant assignment algorithm performs better than an alternative nearest-neighbor based assignment algorithm^1^.

## 1 Introduction

### 1.1 Motivation

Pursuit-evasion is an important problem in robotics with a wide range of applications including environmental monitoring and surveillance. Very often evaders are adversarial agents whose exact locations or actions are not known and can at best be modeled stochastically. Even when the pursuers are more capable and more numerous than the evaders, capture time may be highly unpredictable in such probabilistic settings. Optimization of time-to-capture in presence of uncertainties is a challenging task, and an understanding of how best to make use of the excess resources/capabilities is key to achieving that. This paper address the problem of assignment of pursuers to evaders and control of pursuers under such stochastic settings in order to minimize the expected time to capture.

### 1.2 Problem Overview

We consider a multi-agent pursuit-evasion problem where, in a known environment, we have several surveillance robots (the pursuers) for monitoring a workspace for potential intruders (the evaders). Each evader emits a weak and noisy signal (for example, wifi signal used by the evaders for communication or infrared heat signature), that the pursuers can detect using noisy sensors to estimate their position and try to localize them. We assume that the signals emitted by each evader are distinct and is different from any type of signal that the pursuers might be emitting. Thus the pursuers can not only distinguish between the signals from the evaders and other pursuers, but also distinguish between the signals emitted by the different evaders. Likewise, each pursuer emits a distinct weak and noisy signal that the evaders can detect to localize the pursuers. Each agent is aware of its own location in the environment and the agents of the same type (pursuers or evaders) can communicate among themselves. The environment (obstacle map) is assumed to be known to either type of agents.

**FIGURE 1:**
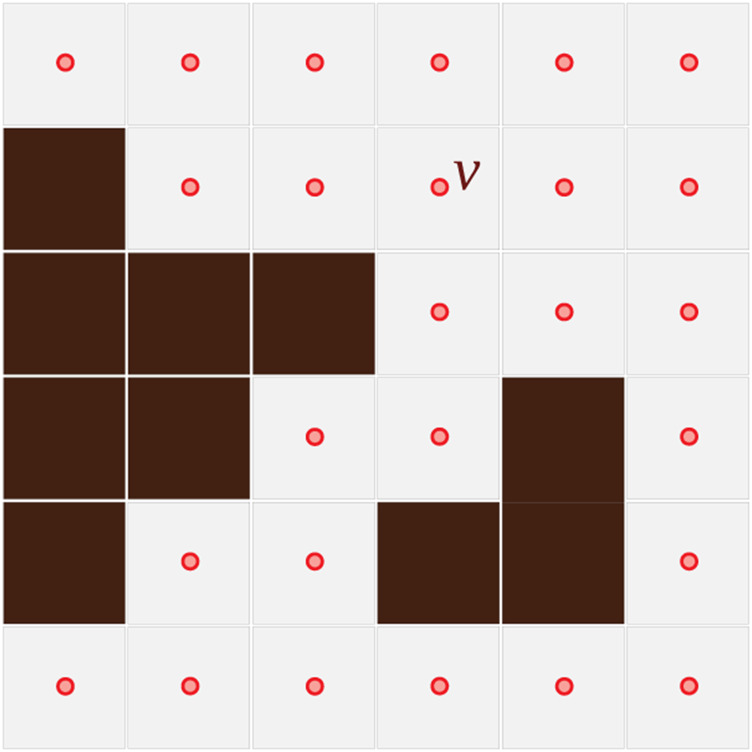
Discrete representation of the planar configuration space, C. The dark brown cells are inaccessible (obstacles), and a vertex corresponds to each accessible cell.

Each evader uses a control strategy that actively avoids the pursuers. The pursuers need to use an assignment strategy and a control strategy that allow them to follow the path with least expected capture time. The evaders and pursuers are aware of each others’ strategies (this, for example, represents real-world scenario where every agent uses an open-source control algorithm), however, the exact locations and actions taken by one type of agent (evader/purser) at an instant of time is not known to the other type (pursuer/evader). Using the noisy signals and probabilistic sensor models, each type of agent maintains and updates (based on sensor measurements as well as the known control/motion strategy) a probability distribution that random variable for evader position type (pursuer/evader) see Figure 1. In this paper we use a first-order dynamics (velocity control) model for point agents (pursuers or evaders) as is typically done in many multi-agent problems such as coverage control (Cortes et al., 2004; Bhattacharya et al., 2014) and artificial potential function based navigation Rimon and Koditschek (1992).

### 1.3 Contributions

The main contributions of this paper are novel methods for pursuer-to-evader assignment in presence of uncertainties for total capture time minimization as well as for maximum capture time minimization. We also present a novel control algorithm for pursuers based on Theta* search (Nash et al., 2007) that takes the evaders’ probability distribution into account, and present a control strategy for evaders that try to actively avoid the pursuers trying to capture it. We assume that both groups of agents (pursuers and evaders) are aware of the control strategies employed by the other group, and can use that knowledge to predict and update the probability distributions that are used for internal representations of the competing group.

### 1.4 Overview of the Paper

Section 3 provides the technical tools and background for formally describing the problem. In Section 4, we introduce the control strategies for the evaders and pursuers. In presence of uncertainties this control strategy becomes a stochastic one. We also describe how each type of agent predict and update the probability distributions representing the other type using this known control strategy. In Section 5, we present algorithms for assigning pursuers to the probabilistic evaders so as to minimize the expected time to capture. In Section 6 simulation and comparison results are presented.

## 2 Related Work

The pursuit-evasion problem in a probabilistic setting requires localization of the evaders as well as development of a controller for the pursuer to enable it to capture the evader. Markov localization is an effective approach for tracking probabilistic agents in unstructured environments since it is capable of representing probability distributions more general than normal distributions [unlike Kalman filters (Barshan and Durrant-Whyte, 1995)]. Compared to Monte Carlo or particle filters (Fox et al., 1999a; Fox et al., 1999b), Markov localization is often computationally less intensive, more accurate and has stronger formal underpinnings.

Markov localization has been widely used for estimating an agent’s position in known environments (Burgard et al., 1996) and in dynamic environments (Fox et al., 1999b) using on-board sensors, as well as for localization of evaders using noisy external sensors (Fox et al., 1998; Fox et al., 1999b; Zhang, 2007a). More recently, in conjunction with sensor fusion techniques, Markov localization has been used for target tracking using multiple sensors (Zhang, 2007b; Nagaty et al., 2015).

Detection and pursuit of an uncertain or unpredictable evader has also been studied extensively. (Chung et al., 2011) provides a taxonomy of search and pursuit problems in mobile robotics. Different methods are compared in both graphs and polygonal environments. Under that taxonomy, our work falls under the domain of probabilistic search problems with multiple heterogeneous searchers/pursuers and multiple targets on a finite graph representation of the environment. Importantly, this survey however notes that the minimization of distance and time to capture the evaders is less studied. (Khan et al., 2016) is another comprehensive review focused on cooperative multi-robot targets observation. (Hollinger et al., 2007) describes strategies for pursuit-evasion in an indoor environment which is discretized into different cells, with each cell representing a room. However, in our approach, the environment is discretized into finer grids that generalize to a wider variety of environments. In (Hespanha et al., 1999) a probabilistic framework for a pursuit-evasion game with one evader and multiple pursuers is described. A game-theoretic approach is used in (Hespanha et al., 2000) to describe a pursuit-evasion game in which evaders try to actively avoid the pursuers. (Makkapati and Tsiotras, 2019) describes an optimal strategy for evaders in multi-agent pursuit-evasion without uncertainties. Along similar lines, in (Oyler et al., 2016) the authors describe a pursuit-evasion game in presence of obstacles in the environment. (Shkurti et al., 2018) describes a problem involving a robot that tries to follow a moving target using visual data. Patrolling is another approach to pursuit-evasion problems in which persistent surveillance is desired. Multi-robot patrolling with uncertainty have been studied extensively in (Agmon et al., 2009), (Agmon et al., 2012) and (Talmor and Agmon, 2017). More recently in (Shah and Schwager, 2019), Voronoi partitioning has been used to guide pursuers to maximally reduce the area of workspace reachable by a single evader. Voronoi partitioning along with area minimization has also been used for pursuer-to-evader assignments in problems involving multiple deterministic and localized evaders and pursuers (Pierson et al., 2017).

## 3 Problem Formulation

### 3.1 Representing the Pursuers, Evaders, and Environment

Since the evaders are represented by probability distributions by the pursuers, the time-to-capture an evader by a particular pursuer is a stochastic variable. We thus consider the problems of pursuer-to-evader assignment and computation of control velocities for the pursuers with a view of minimizing the total expected capture time (the sum of the times taken to capture each of the evaders) or the maximum expected capture time (the maximum out of the times taken to capture each of the evaders). We assume that the number of pursuers is greater that the number of evaders and that the pursuers constitute a heterogeneous team, with each having different maximum speeds and different capture capabilities. The speed of the pursuers are assumed to be higher than the evaders to enable capture in any environment (even obstacle-free or unbounded environment). The objective of this paper is to design strategies for the pursuers to assign themselves to the evaders, and in particular, algorithms for assignment of the excess (redundant) pursuers, so as to minimize the total/maximum expected capture time.

While the evaders know the pursuers’ assignment strategy, they don’t know the pursuers’ positions, the probability distributions that the pursuers use to represent the evaders, or the exact assignment that the evaders determine. Instead, the evaders rely on the probability distributions that they use to represent the pursuers to figure out the assignments that the pursuers are likely using. We use a Markov localization (Thrun et al., 2005) technique to update the probability distribution of each agent.

Throughout this paper we use the following notations to represent the agents and the environment:

Configuration Space Representation: We consider a subset of the Euclidean plane, $C\subset R^{2}$, as the configuration space for the pursuers as well as the evaders, which we discretize into a set of cells or vertices, *V*, where the agents can reside (Figure 1). A vertex in *V* will be represented with a lower-case letter *v* ∈ *V*, while its physical position (Euclidean coordinate vector) in *C* will be represented as **X**(*v*). For simplicity, we also use a discrete time representation.

Agents: The *i* ^th^ pursuer’s location is represented by *r*_*i*_ ∈ *V*, and the *j* ^th^ evader by *y*_*j*_ ∈ *V* (we will use the same notations to refer to the respective agents themselves). The set of the indices of all the pursuers is denoted by $C_{r}$, and the set of the indices of all the evaders by $C_{y}$.

Heterogeneity: Pursuer *r*_*i*_ is assumed to have a maximum speed of *v*_*i*_, and the objective being time minimization, it always maintains that highest possible speed. It also has a capture radius (i.e., the radius of the disk within which it can capture an evader) of *ρ*_*i*_.

### 3.2 Probabilistic Representations

**FIGURE 2:**
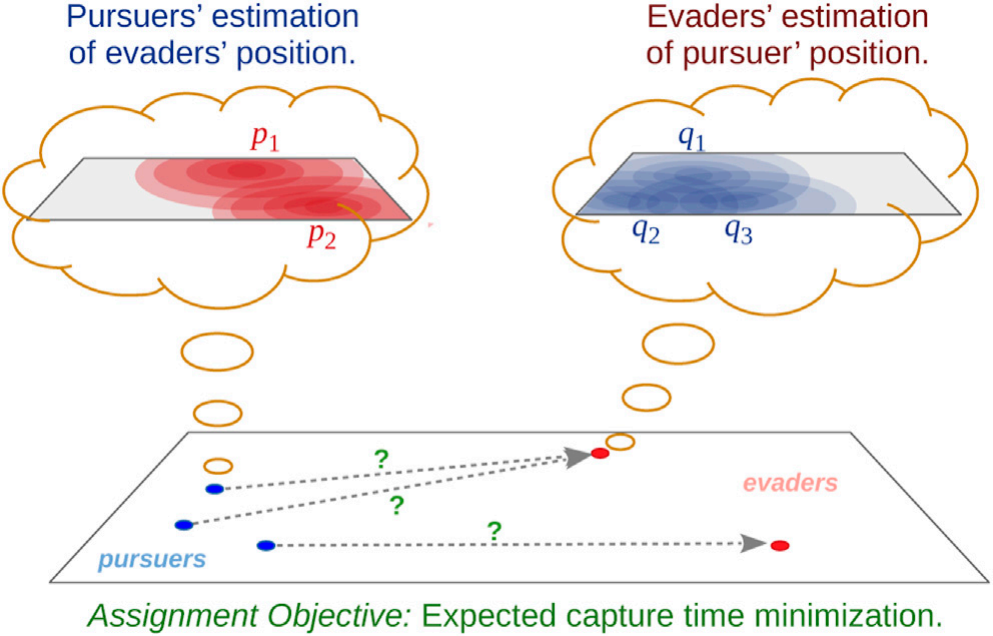
Problem overview.

The pursuers represent the *j* ^th^ evader by a probability distribution over *V* denoted by $p_{j}^{t}:V\to R_{+}$. Likewise the evaders represent the *i* ^th^ pursuer by a probability distribution over *V* denoted by $q_{i}^{t}:V\to R_{+}$. The pursuers maintain the evader distributions, ${\left\{p_{j}^{t}\right\}}_{j\in C_{y}}$, which are unknown to the evaders. While the evaders maintain the pursuer distributions, ${\left\{q_{i}^{t}\right\}}_{i\in C_{r}}$, which are unknown to the pursuers (see Figure 2). The superscript *t* emphasizes that the distributions are time-varying since they are updated by each type of agent (pursuer/evader) based on known control strategy of the other type of agent (evader/pursuer) and models for sensors on-board the agents.

#### 3.2.1 Motion Model

At every time-step the known control strategy (hence, known transition probabilities) allows one type of agent to predict the probability distribution of the other type of agent in the next time-step:

$$\begin{array}{l} \text{Pursuer’s estimation of evader’s position}\left(\mathrm{prediction step}\right):{\tilde{p}}_{j}^{t}\left(y\right)=\underset{y^{\prime }\in V}{\sum }K_{j}\left(y,y^{\prime }\right)\;p_{j}^{t-1}\left(y^{\prime }\right)\\ \text{Evader’s estimation of pursuer’s position}\left(\mathrm{prediction step}\right):{\tilde{q}}_{i}^{t}\left(r\right)=\underset{r^{\prime }\in V}{\sum }L_{i}\left(r,r^{\prime }\right)\;q_{i}^{t-1}\left(r^{\prime }\right) \end{array}$$ (1)

where using the first equation the pursuers predict the *j* ^th^ evader’s probability distribution at the next time-step using the transition probabilities *K*_*j*_ computed using the known control strategy of the evader. While the second equation is used by the evaders to predict the *i* ^th^ pursuer’s probability distribution using transition probabilities *L*_*i*_ computed from the known control strategy of the pursuers. These control strategies and the resulting transition probabilities will be discussed in more details in Sections 4.1 and 4.2.

#### 3.2.2 Sensor Model

**FIGURE 3:**
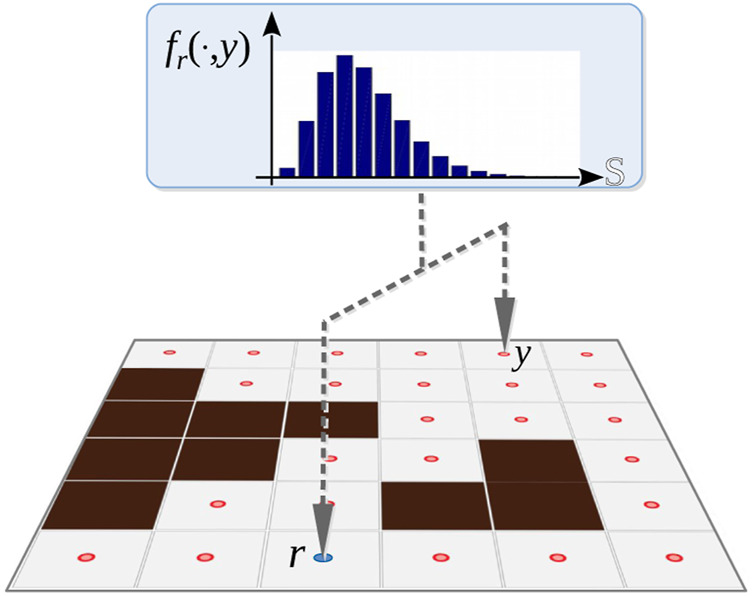
For fixed *r*, *y*, the plot shows the probability distribution over the signal space S.

We assume that the probability that a pursuer at *r* ∈ *V* measures signal *s* (in some discrete signal space S) using its on-board sensors if the evader is at *y* ∈ *V* is given by the probability distribution $f_{r}:S\times V\to R_{+}$, $f_{r}\left(s,y\right)\;\;=\;\;P\left(S=s|Y=y\right)$ where, S is the random variable for signal measurement, and Y is the random variable for evader position (see Figure 3). Likewise, $h_{y}\left(s,r\right)=P\left(S=s|R=r\right)$ is the senor model used by the evaders giving the probability that an evader at *y* measures signal *s* when a pursuer is at *r*.

Using Bayes’ rule, the updated probability distribution of the *j* ^th^ evader as computed by a pursuer at, *r*, based on sensor *measurement*, *s* ^*t*^, and the prior probability estimate, ${\tilde{p}}_{j}^{t}$, is

$$p_{j}^{t}\left(y\right)=P\left(Y_{j}=y|S_{j}=s^{t}\right)=P\left(S_{j}=s^{t}|Y_{j}=y\right)\;\frac{P\left(Y_{j}=y\right)}{P\left(S_{j}=s^{t}\right)}\;\;=\;\;\frac{f_{r}\left(s^{t},y\right)\;{\tilde{p}}_{j}^{t}\left(y\right)}{\sum_{y^{\prime }\in V}f_{r}\left(s^{t},y^{\prime }\right)\;{\tilde{p}}_{j}^{t}\left(y^{\prime }\right)}$$

If multiple signals, $s_{1}^{t},s_{2}^{t},\cdots \;$, are received by pursuers *r*_1_, *r*_2_, ⋯ at a time step, they are incorporated in sequence:

$$\text{Pursuer’s estimation of evader’s postion}\left(\mathrm{update step}\right):\;p_{j}^{t}\left(y\right)=\underset{l}{\prod }\frac{f_{r_{l}}\left(s_{l}^{t},y\right)}{\sum_{y^{\prime }\in V}f_{r_{l}}\left(s_{l}^{t},y^{\prime }\right)\;{\tilde{p}}_{j}^{t}\left(y^{\prime }\right)}\;{\tilde{p}}_{j}^{t}\left(y\right)$$ (2)

Likewise, the evaders *y*_1_, *y*_2_, ⋯ measuring signals $s_{1}^{t},s_{2}^{t},\cdots \;$ update the probability distributions that they use to represent the *i* ^th^ pursuer according to

$$\text{Evader’s estimation of pursuer’s postion}(\mathrm{update step}):\;q_{i}^{t}\left(r\right)=\underset{l}{\prod }\frac{h_{y_{l}}\left(s_{l}^{t},r\right)}{\sum_{r^{\prime }\in V}h_{y_{l}}\left(s_{l}^{t},r^{\prime }\right)\;{\tilde{q}}_{i}^{t}\left(r^{\prime }\right)}\;{\tilde{q}}_{i}^{t}\left(r\right)$$ (3)

The specific functional form for *f* and *h* depend not only on the distance between the pursuers and the evaders in the environment, but also on the obstacles that results on degradation of the signals emitted by the agents. The details of the specific sensor models appear in the “Results” section (Section 6).

### 3.3 Assignment Fundamentals

The goal for our assignment strategy is to try to find the assignment that minimizes either the total expected capture time (the sum of the times taken to capture each of the evaders in $C_{y}$) or the maximum expected capture time (the maximum out of the times taken to capture each of the evaders in $C_{y}$). We assume that there are more pursuers in the environment than the number of evaders. The following subsection provides some fundamental definitions and tools that are used to describe and solve the optimal assignment problem in Section 5.

#### 3.3.1 Formal Description of Assignment

In order to formally describe the assignment problem, we use the following notations:

Assignment: The set of pursuers assigned to the *j* ^th^ evader will be represented by the set *I*_*j*_. The individual assignment of *i* ^th^ pursuer to *j* ^th^ evader will be denoted by the pair (*i*, *j*). $F=\left\{\left(i,j\right)|i\in C_{r},j\in C_{y}\right\}$ denotes the set of all possible such pursuer-to-evader pairings.

A (valid) assignment, A⊆F, is such that for every $\left(i,j\right),\left(i^{\prime },j^{\prime }\right)\in A$, we should have *i* = *i*′ ⇒ *j* = *j*′ (i.e., a pursuer cannot be assigned to two different evaders). This also implies $|\left\{j\;|\;\left(i,j\right)\in A\right\}|\leq 1,\;\forall i\in C_{r}$ (note that an assignment allows for unassigned pursuers).

The set of all possible valid assignments is denoted by $A=\left\{A\subseteq F\;|\;\forall \left(i,j\right),\left(i^{\prime },j^{\prime }\right)\in A,\;i\;=\;i^{\prime }\Rightarrow j\;=\;j^{\prime }\right\}$.

#### 3.3.2 Probabilistic Assignment Costs

In this section we consider the time that pursuer *i* takes to capture evader *j*. We describe the computation from the perspective of the pursuers. Since the evader *j* is represented by the probability distribution, *p*_*j*_, over *V*, we denote *T*_*ij*_ as the random variable representing the uncertain travel time from pursuer *i* to evader *j*. The probability that *T*_*ij*_ falls within a certain interval is the sum of all the probabilities on the vertices of *V* such that the travel time from *r*_*i*_ to the vertex is within that interval. That is,

$$P\left(T_{ij}\in \left[\tau ,\tau +\Delta \tau \right)\right)=\underset{\left\{y\;\in \;V\;|\frac{1}{v_{i}}d_{g}\left(r_{i},y\right)\in \left[\tau ,\tau +\Delta \tau \right)\right\}}{\sum }\;\;\;\;p_{j}\left(y\right)$$

We first note that *T*_*ij*_ and *T*_*ij*′_ are independent variables whenever *j* and *j*′ are different (i.e., the time taken to reach evader *j* does not depend on time taken to reach evader *j*′). However, *T*_*ij*_ and *T*_*i*′*j*_ are dependent random variables since, for a given travel time (and hence travel distance) from pursuer *i* to evader *j*, and knowing the distance between pursuers *i* and *i*′, the possible values of distances between pursuer *i*′ and evader *j* are constrained by the triangle inequality. That is, for any given *j*, the random variables in the set $\left\{T_{ij}\;|\;i\in I\right\}$, where I is a set of pursuer indices, are dependent. This can be seen more clearly by considering a potential evader position *y* ∈ *V* which has an associated probability of *p*_*j*_(*y*). Given that position, $\frac{1}{v_{i}}d_{g}\left(y,r_{i}\right)$ is the time taken by the pursuer i∈I to reach the evader. In particular, the following holds:

$$\begin{array}{ccc} & & P\left(\underset{i\in I}{⋀}\;T_{ij}\in \left[\tau_{i},\tau_{i}+\Delta \tau_{i}\right)\right)\\ & = & \underset{y}{\sum }P\left(\underset{i\in I}{⋀}\frac{1}{v_{i}}d_{g}\left(r_{i},y\right)\in \left[\tau_{i},\tau_{i}+\Delta \tau_{i}\right)\right)\\ & = & \underset{\left\{y\in V\;|\;\frac{d_{g}\left(r_{i},y\right)}{v_{i}}\;\in \;\left[\tau_{i},\tau_{i}+\Delta \tau_{i}\right),\forall i\in I\right\}}{\sum }p_{j}\left(y\right) \end{array}$$ (4)

Thus, in order to compute the joint probability distributions of $\left\{T_{ij}\;|\;i\in I\right\}$, we can sample a *y* from the probability distribution *p*_*j*_ and compute the travel times $\tau_{i}=\frac{1}{v_{i}}d_{g}\left(r_{i},y\right),\;i\in I$, and hence populate the distribution.

### 3.4 Problem Objectives

In the next sections we will describe the control strategy used by a pursuer that allows it to effectively capture the evader assigned to it, as well as the control strategy of an evader that allows it to move away from the pursuers assigned to it.

In Section 5, for designing the assignment strategy for the pursuers we will consider two metrics to minimize: 1) the total expected capture time, which is the sum of the times taken to capture each of the evaders, and, 2) the maximum expected capture time, which is the times taken to capture the last evader. While the actual assignment is computed by the pursuers and unavailable to the evaders, the evaders will estimate the likely assignment in order to determine their control strategy.

As mentioned earlier, we assume that both types of agents know all the strategies used by the other type of agents. That is, the pursuers know the evaders’ control strategy and the evaders know the pursuers’ control and assignment strategies. However the pursuers do not know the evaders’ exact position and vice versa. Instead they reason about that by maintaining probability distributions representing the positions of the other type of agents and update those distributions using the known control strategies of the other type of agents and weak signals measured by onboard sensors.

## 4 Control Strategies

Assuming a known pursuer-to-evader assignment, in this section we describe the control strategies used by the evaders to avoid being captured and the control strategy used by the pursuers to capture the evaders.

### 4.1 Evader Control Strategy

In presence of pursuers, an evader *y*_*j*_ actively tries to move away from the pursuers targeting it. With the evader at *y* ∈ *V* and deterministic pursuers, ${\left\{r_{i}\right\}}_{i\in I_{j}}$, trying to capture it, we define a mean capture time as the harmonic mean of the capture time for each of the pursuers:

$$\tau \left(y,{\left\{r_{i}\right\}}_{i\in I_{j}}\right)\;=\;\frac{1}{\sum_{i\in I_{j}}\frac{1}{{\tilde{d}}_{g}\left(r_{i},y\right)/v_{i}}}$$ (5)

where ${\tilde{d}}_{g}\left(r_{i},y\right)=\max\left(0,\;d_{g}\left(r_{i},y\right)-\rho_{i}\right)$ is the effective geodesic distance between *r*_*i*_, *y* ∈ *V* (with *d*_*g*_(*r*_*i*_, *y*) being the geodesic distance or shortest path length between *r*_*i*_ and *y*), which accounts for the fact that pursuer *r*_*i*_ has a capture radius of *ρ*_*i*_. For a given set of pursuer positions, *τ* thus a function that has higher value on the vertices in *V* that are farther away from the pursuers in *I*_*j*_. The reason behind taking harmonic mean is that the harmonic mean gets lower contribution from distant pursuers and higher contribution from the nearby pursuers.

**FIGURE 4:**
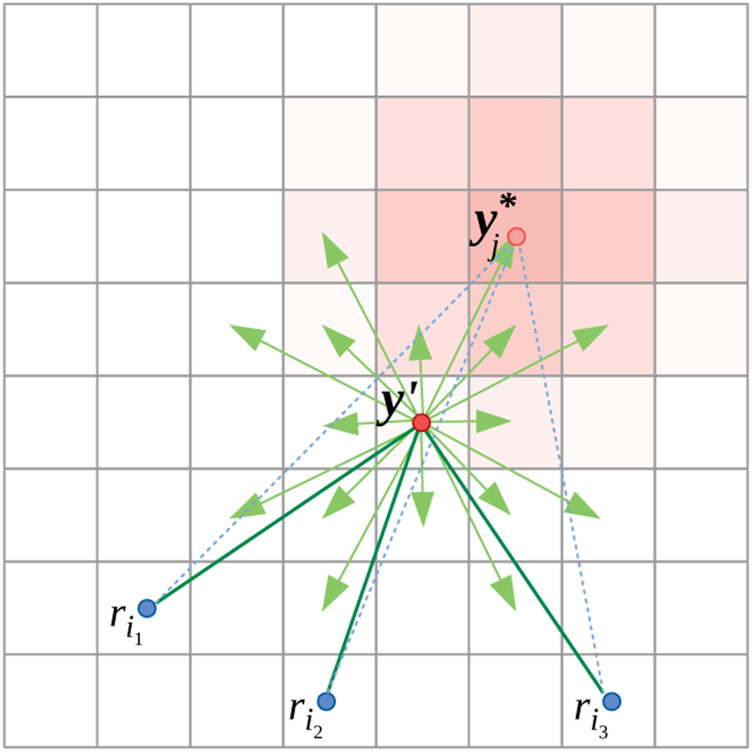
Illustration of control strategy of evader at *y*′. Transition probabilities, *K*_*j*_ (⋅, *y*′) are shown in light red shade.

In order to determine the best action that the evader at *y*′ ∈ *V* can take, it computes the marginal increase in *τ* if it moves to *y* ∈ *V* (Figure 4):

$$\Delta \tau \left(y,y^{\prime },{\left\{r_{i}\right\}}_{i\in I_{j}}\right)\;=\;\max\left(0,\tau \left(y,{\left\{r_{i}\right\}}_{i\in I_{j}}\right)\;-\;\tau \left(y^{\prime },{\left\{r_{i}\right\}}_{i\in I_{j}}\right)+\epsilon \right)$$ (6)

where *ϵ* is a small number that gives a small positive marginal increase for some neighboring vertices in scenarios when the evader gets cornered against an obstacle.

#### 4.1.1 Evader’s Control Strategy

In a deterministic setup the evader at *y*′ will move to

$$y_{j}^{*}\left(y^{\prime },{\left\{r_{i}\right\}}_{i\in I_{j}}\right)\;≔\;\arg\underset{y\in A_{y^{\prime }}}{\max}\Delta \tau \left(y,y^{\prime },{\left\{r_{i}\right\}}_{i\in I_{j}}\right)$$ (7)

where *A*_*y*′_ refers to the states/vertices in the vicinity of *y*′ that the evader can transition to in the next time-step. But, in the probabilistic setup where the evaders represent the *i* ^th^ pursuer by the distribution *q*_*i*_, with every *y* ∈ *A*_*y*′_ an evader associates a probability that it is indeed the best transition to make. In practice, these probabilities are computed by sampling ${\left\{r_{i}\right\}}_{i\in I_{j}}$ from the distributions ${\left\{q_{i}\right\}}_{i\in I_{j}}$, and counting the proportion of samples for which a *y* ∈ *A*_*y*′_ is the neighbor that maximizes the marginal increase in capture time. The evader then uses this probability distribution over its neighboring states to make a stochastic transition.

#### 4.1.2 Pursuer’s Prediction of Evader’s Distribution Based on Known Evader Control Strategy

The pursuers know the evader’s strategy of maximizing the marginal increase in capture time. However, they do not know the evaders’ exact position, nor do they know the distributions, *q*_*i*_, that the evaders maintain of the pursuers. The uncertainty in the action of the evader due to that is modeled by a normal distribution centered at $y_{j}^{*}\left(y^{\prime },{\left\{r_{i}\right\}}_{i\in I_{j}}\right)$. If the evader is at *y*′, the transition probability *K*_*j*_ (*y*, *y*′) is the assumed to be

$$K_{j}\left(y,y^{\prime }\right)=\left\{\begin{array}{cc} \kappa_{j}\exp\left(-\frac{d_{f}{\left(y,\;y_{j}^{*}\left(y^{\prime },{\left\{r_{i}\right\}}_{i\in I_{j}}\right)\right)}^{2}}{2\sigma_{j}^{2}}\right)\;\;,\; & \text{if}y\in A_{y^{\prime }}\\ 0,\; & \text{otherwise.} \end{array}\right.$$ (8)

where, for simplicity, *d*_*f*_ is assumed to be the Euclidean distance between the neighboring vertices in the graph, and *κ*_*j*_ is a normalization factor so that *∑*_*y*∈*V*_ *K*(*y*, *y*′) = 1.

### 4.2 Pursuer Control Strategy

A pursuer, *r*_*i*_ ∈ *I*_*j*_, pursuing the evader at *y*_*j*_ needs to compute a velocity for doing so.

**FIGURE 5:**
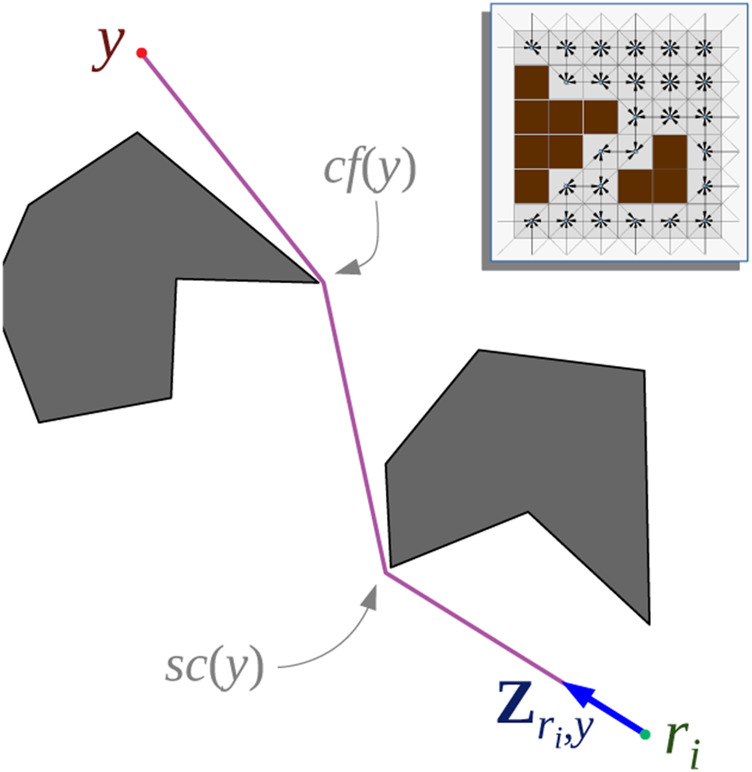
Theta* algorithm is used on a 8-connected grid graph, *G*_✳_ (top right inset) for computing geodesic distances as well as control velocities for the pursuers.

In a deterministic setup, if the evader is at *y*_*j*_ ∈ *V*, the pursuer’s control strategy is to follow the shortest (geodesic) path in the environment connecting *r*_*i*_ to *y*_*j*_. This controller, in practice, can be implemented as a gradient-descent of the square of the path metric (geodesic distance) and is given by $v_{i}=k\frac{\partial d_{g}{\left(r_{i},y_{j}\right)}^{2}}{\partial X\left(r_{i}\right)}=2k\;d_{g}\left(r_{i},y_{j}\right)\;{\hat{z}}_{r_{i},y_{j}}$, where *k* is a proportionality constant, *d*_*g*_ (*r*_*i*_, *y*_*j*_) is the shortest path (geodesic) distance between *r*_*i*_ and *y*_*j*_, and ${\hat{z}}_{r_{i},y_{j}}$ is the unit vector to the shortest path at *r*_*i*_ (see Figure 5). Such a controller does not suffer from local minimas due to presence of non-convex obstacles since the geodesic paths *go around* obstacles. A formal proof of that and the fact that $\frac{\partial d_{g}\left(r,y\right)}{\partial X\left(r\right)}={\hat{z}}_{r,y}$, appeared in (Bhattacharya et al., 2014).). This gives a simple velocity controller for the pursuer.

#### 4.2.1 Pursuer’s Control Strategy

Since the pursuers describe the *j* ^th^ evader’s position by the probability distribution $p_{j}^{t}$ over *V*, we compute an expectation on the velocity vectors of the *i* ^th^ pursuer (with *i* ∈ *I*_*j*_) as follows:

$${\hat{v}}_{i}=\underset{y\in V}{\sum }2k\;d_{g}\left(r_{i},y\right)\;{\hat{z}}_{r_{i},y}\;p_{j}^{t}\left(y\right)$$ (9)

Since the pursuer has a maximum speed of *v*_*i*_, and the exact location of the evader is unknown, we always choose the maximum as speed for the pursuer: $v_{i}\;=\;v_{i}\;\frac{{\hat{v}}_{i}}{\left\|{\hat{v}}_{i}\right\|}$.

For computing *d*_*g*_ (*r*_*i*_, *y*) we use the Theta* search algorithm (Nash et al., 2007) on a uniform 8-connected square grid graph, *G*_✳_, representation of the environment (Figure 5 inset). While very similar to Dijkstra’s and A*, Theta* computes paths that are not necessary restricted to the graph and are closer to the true shortest path in the environment. While more advanced variations of the algorithm exists [such as Lazy Theta* (Nash et al., 2010) and Incremental Phi* (Nash et al., 2009)], we choose to use the most basic variety for simplicity. Computation of the sum in Equation 9 can also be performed during the Theta* search. Algorithm 1 describes the computation of *d*_*g*_ (*r*_*i*_, *y*) (the shortest path (geodesic) distance between *r*_*i*_ and a point *y* in the environment) and the control velocity **v**_*i*_.

**Algorithm 1:** Theta* Based Pursuer Control

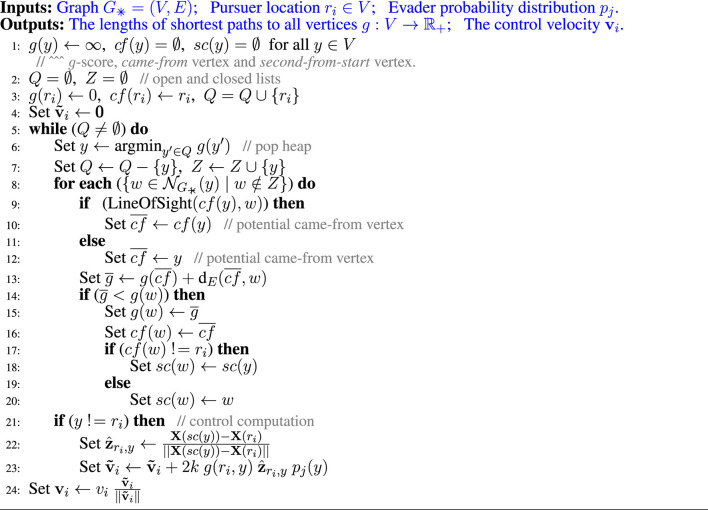

The algorithm is reminiscent of Dijkstra’s search, maintaining an open list, *Q*, and expanding the least *g*-score vertex at every iteration, except that the came-from vertex (*cf*) of a vertex can be a distant predecessor determined by line of sight (Lines 10–14) and the summation in (9) is computed on-the-fly during the execution of the search (Line 28).

We start the algorithm by initiating the open list with the single start vertex, *r*_*i*_, set its *g*-score to zero, and its came-from vertex, *c f*, to reference to itself (line 4). Every time a vertex, *y* (one with the minimum *g*-score in the open list, maintained using a heap data structure), is expanded, Theta* checks for the possibility of updating a neighbor, *w*, from the set of neighbors, $N_{G_{+\;\times }}\left(y\right)$, of the vertex that are not in the closed list (line 9). Based on the existence of a direct line of sight from the came-from vertex of *y* and the vertex *w*, the potential new came-from vertex, $\bar{cf}$, is set to *cf*(*y*) or *y*. The new potential *g*-score is computed as the sum of the *g*-score of $\bar{cf}$ and the Euclidean distance, ${\text{d}}_{E}\left(\bar{cf},w\right)=\|X\left(\bar{cf}\right)-X\left(w\right)\|$, between the two vertices. If lower, *g*(*w*) is updated, the came-from vertex of *w* is set to $\bar{cf}$, and the vertex on the path second from the start, *sc*(*w*), is copied from that of *y* unless *w* is itself second from start. We also compute the control velocity as part of the Theta* search algorithm. Every time a vertex is expanded, we add the corresponding term in the summation of Equation 9 to the vector ${\hat{v}}_{i}$ (line 28), which we scale to have magnitude of the maximum possible speed of the pursuer, *v*_*i*_, at the end.

#### 4.2.2 Evader’s Prediction of Pursuer’s Distribution Based on Known Pursuer Control Strategy

Since the evaders represent the *i* ^th^ pursuer using the probability distribution *q*_*i*_, they need to predict the pursuer’s probability distribution in the next time step knowing the pursuer’s control strategy. This task is assigned to the *j* ^th^ evader such that *i* ∈ *I*_*j*_ (we define $\bar{j}\left(i\right)$ to be the index of the evader assigned to pursuer *i*). It executes Theta* algorithm, similar to Algorithm 1, but the start vertex being *y*_*j*_. Once executed, the line segment connecting any *r*′ ∈ *V* and *cf* (*r*′) gives the direction in which the *i* ^th^ pursuer at *r*′ would tentatively move in the next time-step based on the aforesaid control strategy of the pursuer. Knowing the speed of a pursuer, the evader can thus compute the next position of the pursuer, $r_{j}^{*}\left(r^{\prime },y_{j}\right)$, if it is currently at *r*′. However, in order to account for the fact that the pursuer does not precisely know the evader’s position (and instead use the distribution *p*_*j*_ to represent it), analogous to (8), we use the following transition probability for the prediction step of updating *q*_*i*_

$$L_{i}\left(r,r^{\prime }\right)=\left\{\begin{array}{cc} \kappa_{i}\exp\left(-\frac{d_{f}{\left(r,\;r_{j}^{*}\left(r^{\prime },y_{\bar{j}\left(i\right)}\right)\right)}^{2}}{2\sigma_{i}^{2}}\right),\; & \text{if}r\in A_{r^{\prime }}\\ 0,\; & \text{otherwise.} \end{array}\right.$$ (10)

where *κ*_*i*_ is the normalization factor.

## 5 Assignment Strategies

We first consider the assignment problem from the perspective of the pursuers—with the evaders represented by probability distributions ${\left\{p_{j}\right\}}_{j\in C_{y}}$, what’s the best pursuer-to-evader assigment? In a probabilistic setup, where the costs (capture times) are stochastic variables (see Section 3.3.2), and there are excess pursuers, this needs to be solved in two stages (Prorok, 2020): First we need to determine an initial assignment of each evader to one pursuer. Following that we determine the assignment of the remaining (redundant) pursuers so as to minimize the (total or maximum) expected capture time.

### 5.1 Expected Capture Time Minimization for an Initial One-To-One Assignment

In order to determine an *initial* assignment $A_{0}\subseteq F$ such that exactly one pursuer is assigned to an evader (thus potentially allowing unassigned pursuers).

Since for every $\left(i,j\right),\left(i^{\prime },j^{\prime }\right)\in A_{0}$, *T*_*ij*_ and *T*_*i*′*j*′_ are independent variables, the problem of finding the optimal initial assignment that minimizes the *total expected capture time* becomes^2^

$$\begin{array}{cc} \;\;\;\;A_{0} & =\underset{\begin{array}{c} A^{\prime }\subset F\;\;\text{s.t.}\\ \left(i,j\right),\left(i^{\prime },j^{\prime }\right)\in A^{\prime }\;\Rightarrow \;i\neq i^{\prime },j\neq j^{\prime } \end{array}}{\mathrm{arg min}}E\left(\underset{\left(i,j\right)\in A^{\prime }}{\sum }T_{ij}\right)\\ & =\underset{\begin{array}{c} A^{\prime }\subset F\;\;\text{s.t.}\\ \left(i,j\right),\left(i^{\prime },j^{\prime }\right)\in A^{\prime }\;\Rightarrow \;i\neq i^{\prime },j\neq j^{\prime } \end{array}}{\mathrm{arg min}}\underset{\left(i,j\right)\in A^{\prime }}{\sum }E\left(T_{ij}\right)\;\; \end{array}$$ (11)

Thus, for computing the initial assignment, it is sufficient to use the numerical costs of $C_{ij}=E\left(T_{ij}\right)$ in the assignment of pursuer *i* to evader *j*, and thus find an assignment that minimizes the net cost. In practice we use a Hungarian algorithm to compute the assignment. While a Hungarian algorithm is an efficient method for computing the assignment that minimizes the expected total time of capture, generalizing it to the problem of minimizing the expected maximum capture time is non-trivial, which we address next.

#### 5.1.1 Modified Hungarian Algorithm for Minimization of Maximum Capture Time

For finding the initial assignment that minimizes the *maximum expected capture time*, we develop a modified version of the Hungarian algorithm. To that end we observe that in a Hungarian algorithm, instead of using the expected capture times as the costs, we can use the *p*-th powers of the expected capture times, $C_{ij}={\left(E\left(T_{ij}\right)\right)}^{p}$. Making *p* → *∞* results in the appropriate cost that makes the Hungarian algorithm compute an assignment that minimize the maximum expected capture time (the infinity norm). However, for computation we cannot practically raise a number to infinity, and thus need to modify the Hungarian algorithm at a more fundamental level.

In a simple implementation of the Hungarian algorithm (Munkres, 1957), one performs multiple row and column operations on the cost matrix wherein a specific element of the cost matrix, *C*_*i*′*j*′_, is added or subtracted from all the elements of a selected subset of rows and columns. Thus, if we want to use the *p*th powers of the costs, but choose to maintain only the costs in the matrix (without explicitly raising them to the power of *p* during storage), for the row/column operations we can simply raise the elements of the matrix to the power of *p* right before the addition/subtraction operations, and then take the *p*th roots of the results before updating the matrix entries. That is, addition of *C*_*i*′*j*′_ to an element *C*_*ij*_ will be replaced by the operation $C_{ij}{⊕}_{p}C_{i^{\prime }j^{\prime }}=\sqrt[{p}]{C_{ij}^{p}+C_{i^{\prime }j^{\prime }}^{p}}$, and subtraction will be replaced by the operation $C_{ij}{⊖}_{p}C_{i^{\prime }j^{\prime }}=\sqrt[{p}]{C_{ij}^{p}-C_{i^{\prime }j^{\prime }}^{p}}$.

Thus, letting *p* → *∞*, we have *C*_*ij*_ ⊕_*∞*_ *C*_*i*′*j*′_ = max{*C*_*ij*_, *C*_*i*′*j*′_} and $C_{ij}{⊖}_{\infty }C_{i^{\prime }j^{\prime }}=\left\{\begin{array}{cc} C_{ij},\; & C_{ij}>C_{i^{\prime }j^{\prime }}\\ 0,\; & C_{ij}=C_{i^{\prime }j^{\prime }} \end{array}\right.$. Thus, we can compute the assignment that achieves the minimization of the maximum expected capture time using this modified algorithm, but without actually needing to explicitly raise the costs to the power of a large *p* → *∞*.

### 5.2 Redundant Pursuer Assignment Approach

After computation of an initial assignment, $A_{0}$, we determine the assignment of the remaining pursuers using the method proposed in (Prorok, 2020). Formally, we first consider the problem of selecting a set of redundant pursuer-evader matchings, $\bar{A}$, that minimizes the total expected travel time to evaders, under the constraint that any pursuer is only assigned once:

$$\;\;\;\;\bar{A}=\underset{\begin{array}{c} A^{\prime }\subset F\;\;\text{s.t.}\\ \left(i,j\right),\left(i^{\prime },j^{\prime }\right)\in A^{\prime }\cup A_{0}\;\Rightarrow \;i\neq i^{\prime } \end{array}}{\mathrm{arg min}}\underset{\left(i,j\right)\in A^{\prime }}{\sum }E\left(T_{ij}\right).$$ (12)

Notably, the work in (Prorok, 2020) shows that a cost function such as (12), which considers redundant assignment under uncertain travel time, is supermodular. It follows that the assignment procedure can be implemented with a greedy algorithm that selects redundant pursuers near-optimally^3^.

Algorithm 2 summarizes our greedy redundant assignment algorithm. At the beginning of the algorithm, we sample *h* $\left|C_{r}|\times |C_{y}\right|$-dimensional points from the joint probability distribution of ${\left\{T_{ij}\right\}}_{i\in C_{r},j\in C_{y}}$ and store them in the set $\tilde{T}$. In practice, the sampling is performed by sampling points, *y*_*j*_ ∈ *V*, from the evaders’ probability distributions, *p*_*j*_, for all *j* ∈ *C*_*y*_. The travel times, $\tau_{ij}=\frac{1}{v_{i}}d_{g}\left(r_{i},y_{j}\right),\;i\in C_{r},j\in C_{y}$ then give the sample from the joint probability distributions of ${\left\{T_{ij}\right\}}_{i\in C_{r},j\in C_{y}}$ due to Eq. 4. The *z* ^th^ sample is thus a set of travel times between every pursuer-evader pair, and will be referred to as ${\tilde{T}}^{z}={\left\{\tau_{ij}^{z}\right\}}_{i\in C_{r},j\in C_{y}}\in \tilde{T}$.

**Algorithm 2:** Total Time minimization Redundant Pursuer Assignment

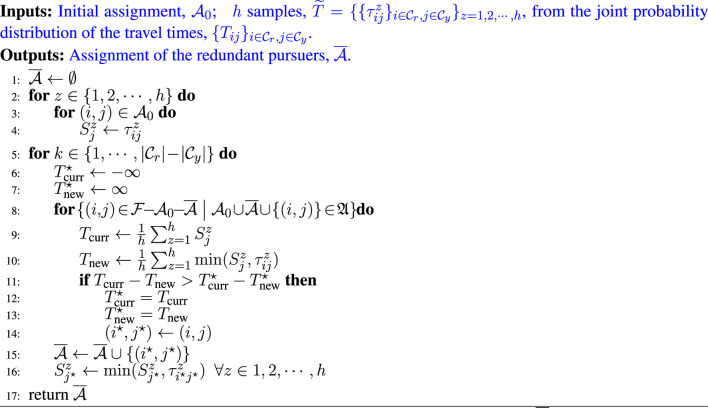

In this algorithm, we first consider the initial assignment, $A_{0}$, and collect all the sampled costs of edges incident on to the *j* ^th^ evader into the variable *S*. Note that a given $j\in C_{y}$ appears in exactly one element of $A_{0}$, thus the assignment in Line 4 assigns a value to a $S_{j}^{z}$ exactly once. The set $\bar{A}$ contains the assignment of the remaining/redundant pursuers, that we initiate with the empty set.

In Line 10, we loop over all the possible pursuer-to-evader pairings, (*i*, *j*), that are not already present in $A_{0}$ or $\bar{A}$, and which, along with $A_{0}$ or $\bar{A}$, constitute a valid assignment. We go through all such potential pairings, (*i*, *j*), and pick the one with the highest marginal gain, $T_{\text{curr}}^{⋆}-T_{\text{new}}^{⋆}$. The pair with the highest marginal gain, is thus added to $\bar{A}$. This process is carried out $\left|C_{r}|\;-\;|C_{y}\right|$ times, thus ensuring that all pursuers get assigned.

### 5.3 Equality in Marginal Gain

One way that the inequality condition in Line 13 gets violated is when the marginal gains *T*_curr_ − *T*_new_ and $T_{\text{curr}}^{⋆}-T_{\text{new}}^{⋆}$ are equal. This can in fact happen quite often when one or more redundant pursuers are left to be assigned and all of them are far from all the evaders, rendering marginal gains for any of the assignments close to zero. In that case a pursuer *i* gets randomly assigned to an evader *j* based on the order in which the pairs $\left(i,j\right)\in F\;-\;A_{0}\;-\;\bar{A}$ are encountered in the for loop of Line 10.

In order to address this issue properly, we maintain a list of “potential assignments” that corresponds to (*i*, *j*) pairs (along with the corresponding *T*_new_ values maintained as an associative list, ${\mathrm{P\backslash relax \backslash special \{t4ht=\}A}}^{⋆}$) that produce the same highest marginal gains (*i.e.*, in line 13 equality holds), and choose the one with the median *T*_new_ value for inserting into the assignment set in Line 19.

#### 5.3.1 Redundant Pursuer Assignment for Minimization of Maximum Capture Time

As for the minimization of the *maximum expected capture time* in the redundant assignment process, we take a similar approach as in Section 5.1.1. We first note that choosing ${\left(E\left(T_{ij}\right)\right)}^{p}$ instead of simply the expected capture time in (12) still keeps the cost function supermodular. If we want to minimize the total (sum) expected *p*th power of the capture time, the condition in the *if* statement in line 13 of the above algorithm needs to be simply changed to $T_{\text{curr}}^{p}-T_{\text{new}}^{p}>{\left(T_{\text{curr}}^{⋆}\right)}^{p}-{\left(T_{\text{new}}^{⋆}\right)}^{p}$. With *p* → *∞*, this condition translates to $\max\left(T_{\text{curr}},T_{\text{new}}^{⋆}\right)>\max\left(T_{\text{curr}}^{⋆},T_{\text{new}}\right)$. Furthermore, to deal with the equality situations in Line 13, instead of choosing the assignment with the median *T*_new_ from ${\mathrm{P\backslash relax \backslash special \{t4ht=\}A}}^{⋆}$, we choose the one with the maximum *T*_new_,

Thus assigning a redundant pursuer to an evader (out of the assignments that produce the same marginal gain) that has the maximum expected capture time, thus providing some extra help with catching the pursuer.

With these modifications, an assignment for the redundant pursuers can be found that minimizes the maximum expected capture time instead of total expected capture time. We call this redundant pursuer assignment algorithm “Maximum Time minimization Redundant Pursuer Assignment” (MTRPA).

### 5.4 Evader’s Estimation of Pursuer Assignment

Knowing the assignment strategy used by the pursuers, but the pursuers represented by the probability distributions ${\left\{q_{i}\right\}}_{i\in C_{r}}$, the evaders use the exact same assignment algorithm to estimate which pursuer is being assigned to it. The only difference is that in Algorithm 2 the elements in the input, $\tilde{T}$, are sample travel times that are computed by sampling points, *r*_*i*_, from the probability distribution, *q*_*i*_, for all $i\in C_{r}$, and then computing $\tau_{ij}=\frac{1}{v_{i}}d_{g}\left(r_{i},y_{j}\right)$ as before. The assignment thus estimated is used by the evaders in computing their control as well as for updating the pursuers’ distributions, ${\left\{q_{i}\right\}}_{i\in C_{r}}$, as described in Sections 4.1.1 and 4.2.2 respectively.

## 6 Results

For the sensor models, *f*, *h*, we emulate sensing electromagnetic radiation in the infrared or radio spectrum emitted by the evaders/pursuers. Wi-fi signals and thermal signatures are such examples. For simplicity, we ignore reflection of the radiation from surfaces, and only consider a simplified model for transmitted radiation. If *I*_*r*,*y*_ is the line segment connecting the source, *y*, of the radiation to the location of a sensor, *r*, and is parameterized by segment length, *l*, we define effective signal distance, $d_{\text{eff}}\left(r,y\right)=\int_{I_{r,y}}\;\rho \left(l\right)\;dl$, where *ρ*(*l*) = 1 in obstacle-free space, and *ρ*_obs_ > 1 inside obstacles to emulate higher absorption of the signal. The signal space, $S=R_{+}$, is the space of intensity of the measured radiation, and *f*_*r*_ and *h*_*y*_ are normal distributions over S with mean $\frac{k_{1}}{d_{\text{eff}}\left(r,y\right)}$ and standard deviation *σ* = *k*_2_ *d*_eff_ (*r*, *y*) to emulate inverse decay of signal strength and higher noise/error for larger separation (we truncate the normal distribution at zero to eliminate negative signal values). In all our experiments we chose *ρ*_obs_ = 3, *k*_1_ = 10. We also fix *k*_2_ = 0.3, except in the experiments in Figure 8, where we evaluate the performance with varying noise level (varying *k*_2_).

The motion models for predicting the probability distributions are chosen as described in Section 4.1.2 and 4.2.2.

For the parameter we choose *ϵ*(*y*) ∈ (0, 0.3) (in Equation 6) depending on whether or not *y* is close to an obstacle. The pursuer (resp. evader) choose *σ*_*j*_ = 0.3 (resp. *σ*_*i*_ = 0.3) for modeling the uncertainties in the evaders’ (resp. pursuers’) estimate of the pursers’ (resp. evaders’) positions.

We Compared the Performance of the Following Algorithms

• Total Time minimizing Pursuer Assignment (TTPA): This assignment algorithm uses the basic Hungarian algorithm for computing the initial assignment $A_{0}$, and uses the TTRPA algorithm (Algorithm 2) for the assignment of the redundant pursuers at every time step. Thus the algorithm seeks to minimize the *total expected capture time* (*i.e.*, sum of the times to capture each evader).
• Maximum Time minimizing Pursuer Assignment (MTPA): This assignment algorithm uses the modified Hungarian algorithm described in Section 5.1.1 for computing the initial assignment $A_{0}$, and uses the MTRPA algorithm (Section 5.3.1) for the assignment of the redundant pursuers at every time step. Thus the algorithm seeks to minimize the *maximum expected capture time* (*i.e.* time to capture the last evader).
• Nearest Neighbor Assignment (NNA): In this algorithm we first construct a $\left|C_{r}|\times |C_{y}\right|$ matrix of expected pursuer-to-evader capture times. An assignment is made corresponding to the smallest element of the matrix, and the corresponding row and column are deleted. This process is repeated until each evader gets a pursuer assigned to it. Then we start the process all over again with the unassigned pursuers and all the evaders, and the process continues until all the pursuers are assigned.

**FIGURE 6:**
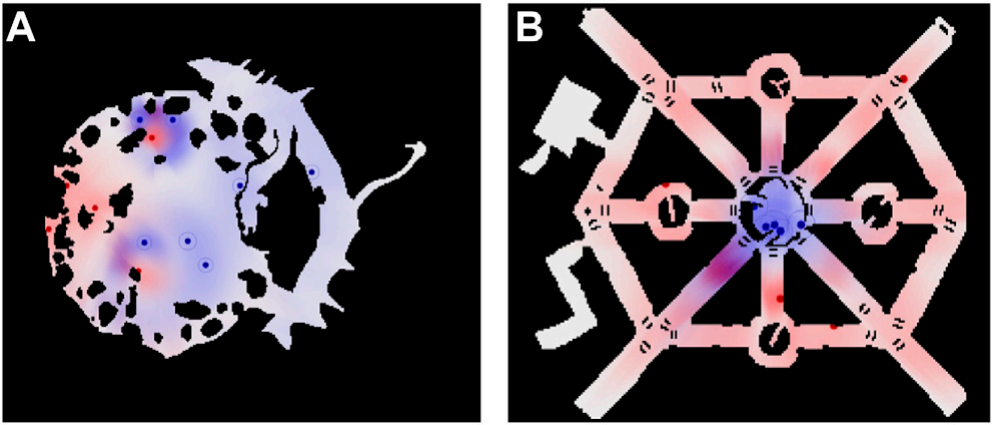
Environments for which statistic are presented. **(A)** “AR0414SR”; **(B)** “AR0701SR.” Each Panel also shows an example of the agent positions and distributions during one of the simulations. Blue hue indicates the evaders’ prediction of pursuers’ distributions, ${\left\{q_{i}\right\}}_{i\in C_{r}}$, while the red hue indicates the pursuers’ prediction of the evaders’ distributions, ${\left\{p_{j}\right\}}_{j\in C_{y}}$.

We evaluated the algorithms in two different environments: Game maps “AR0414SR” and “AR0701SR” from 2D Pathfinding Benchmarks (Sturtevant, 2012) see Figure 6. For different pursuer-to-evader ratios in these environments, we ran 100 simulations each. For each simulation, in environment “AR0414SR”, the initial positions of pursuers and evaders were randomly generated, while in environment :“AR0701SR” the initial position of the pursuers were randomly generated in the small central circular region and the initial position of the evaders were randomly generated in the rest of the environment. For each generated initial conditions we ran the three algorithms, TTPA, MTPA and NNA, to compare their performance.

1) Max capture time in “AR0414SR”
2) Max capture time in “AR0701SR”
3) Total capture time in “AR0414SR”
4) Total capture time in “AR0701SR”

**FIGURE 7:**
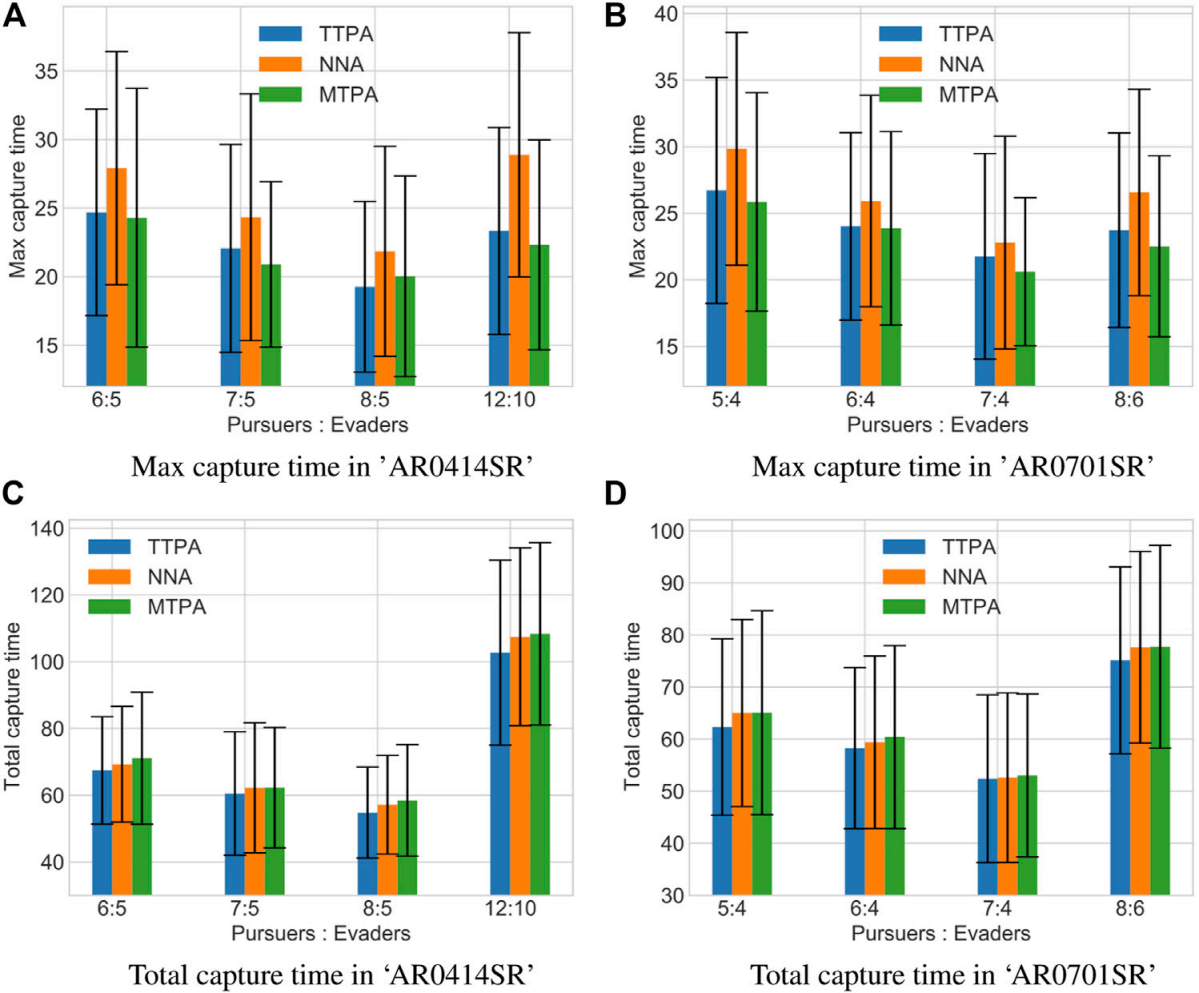
Comparison of the average values of maximum capture times **(A, B)** and total capture times **(C, D)** along with the standard deviation in different environments and with different pursuer-to-evader ratios using the TTPA, NNA and MTPA algorithms. Each bar represents data from 100 simulations with randomized initial conditions.

Figure 7 shows a comparison between the proposed pursuer assignment algorithms (TTPA and MTPA) and the NNA algorithm for the aforementioned environments. From the comparison it is clear that the MTPA algorithm consistently outperforms the other algorithms with respect to the maximum capture time (Figure 7A), while TTPA consistently outperforms the other algorithms with respect to the total capture time (Figure 7C). In addition, Table 1 shows win rates of TTPA and MTPA over NNA (for TTPA this is the proportion of simulations in which the total capture time for TTPA was lower than NNA, while for MTPA this is the proportion of simulations in which the total capture time for MTPA was lower than NNA). TTPA has a win rate of around 60%, and MTPA has a win rate of over 70%.

**TABLE 1:** Win rates of TTPA and MTPA algorithms over NNA. For a given set of initial conditions (initial position of pursuers and evaders), if TTPA takes less total time to capture all the evaders than NNA, it is considered a win for TTPA. While if MTPA takes less time to capture the last evader (maximum capture time) than NNA, it is considered as a win for MTPA.

Algorithm name	AR0414SR (%)	AR0701SR (%)
TTPA	69.3	58.2
MTPA	78.0	71.2

Clearly the advantage of the proposed greedy supermodular strategy for redundant pursuer assignment is statistically significant. Unsurprisingly, we also observe that.increasing the number of pursuers tends to decrease the capture time.

1) Max capture time in “AR0414SR” with 7 pursuers and 5 evaders.
2) Total capture time in “AR0414SR” with 7 pursuers and 5 evaders.

**FIGURE 8:**
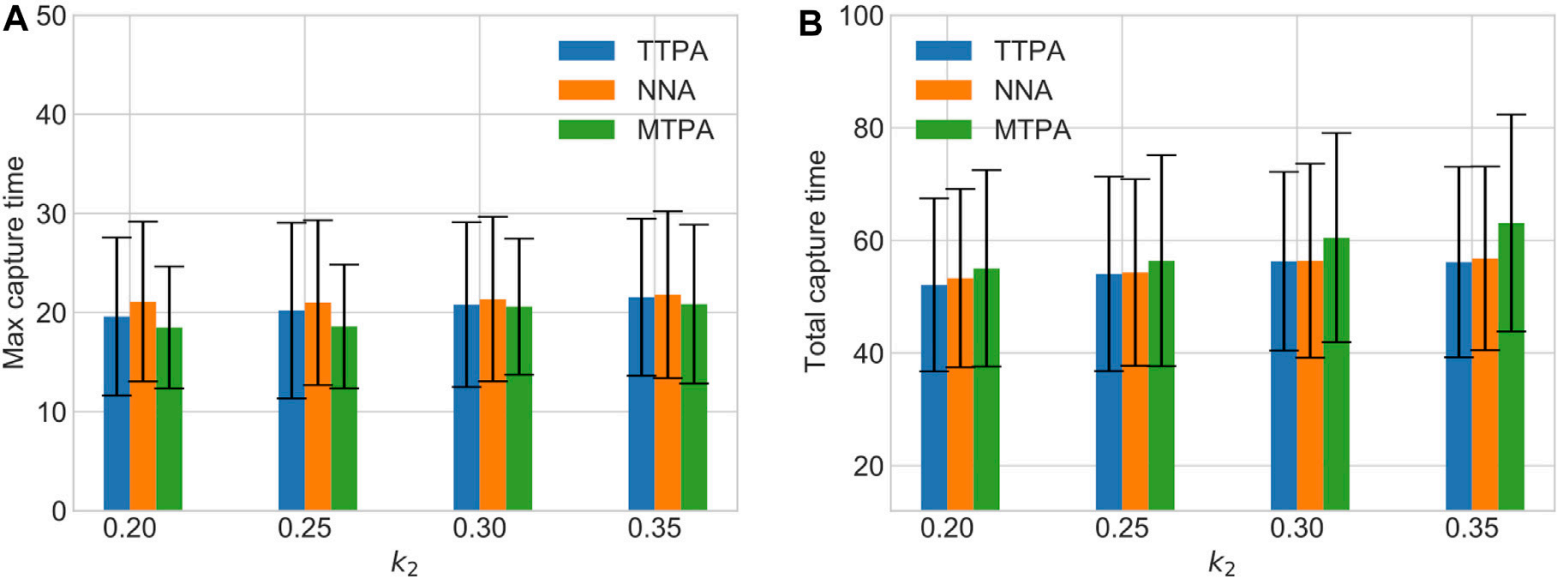
The effect of varying measurement noise level on maximum capture time **(A)** and total capture time **(B)**.

Figure 8 shows a comparison of the total and maximum capture times with varying measurement noise level (varying *k*_2_) in the environment “AR0414SR” with a fixed number of pursuers and evaders, and with 20 randomly generated initial conditions. As expected, higher noise leads to more capture time for all the algorithms. However MTPA still outperforms the other algorithms w.r.t. maximum capture time, while TTPA outperforms the other algorithms w.r.t. the total capture time.

## 7 Conclusion and Discussions

In this paper, we considered a pursuit-evasion problem with multiple pursuers, and multiple evaders under uncertainties. Each type of agent (pursuer or evader) represents the individuals of the other type using probability distributions that they update based on known control strategies and noisy sensor measurements. Markov localization is used to update a probability distributions. The evaders use a control strategy to actively evade the pursuers, while each pursuer use a control algorithm based on Theta* search for reducing the expected distance to the probability distribution of the evader that it’s pursuing. We used a novel redundant pursuer assignment algorithm which utilizes an excess number of pursuers to minimize the total or maximum expected time to capture the evaders. Our simulation results have shown a consistent and statistically significant reduction of time to capture when compared against a nearest-neighbor algorithm.

We considered a very complex problem setup that is not only stochastic in nature (each type of agent representing the other type of agents using probability distributions that are updated using a Markov localization model on a graph), but the environment is non-convex (due to presence of obstacles). While a general stability or convergence guarantee is extremely difficult, if not impossible, in such a complex problem setup, we can consider a simplified scenario for observing some of the stability and convergence properties of the control algorithm used by the pursuers. Such a simplified analysis has been provided in the Appendix below.

## Data Availability

The raw data supporting the conclusions of this article will be made available by the authors, without undue reservation.

## Footnotes

2 The expectation of the sum of two or more independent random variables is the sum of the expectations of the variables

3 We note that without an initial assignment $A_{0}$, any solution that is smaller in size than $\left|C_{y}\right|$ would lead to an infinite capture time, and hence, the cost function looses its supermodular property. Hence, the assumption that we already have an initial assignment is necessary

## Appendix: Simplified Theoretical Analysis

Suppose evader *j* is assigned to pursuer *i* and this assignment does not change. We consider the case when the evader’s maximum speed is negligible compared to the pursuer’s speed, as a consequence of which we make the simplifying assumption that the evader is stationary. The first observation that we can make is that with the stationary evader, the probability distribution for the evader’s pose is updated according to $p_{j}^{t}=D^{t-1}p_{j}^{t-1}$ (see Eq. 2), where $p_{j}^{\tau }$ is a column vector containing the probability values over *V*, and *D* ^*t*−1^ is a diagonal matrix that depends on the signal received as well as the probability distribution at the time-step such that the net probability always adds up to 1. It’s easy to observe that a fixed point of this iteration is a distribution in which all the probability is concentrated on a single vertex, to which the iteration will converge. If the measurement model is unbiased, that vertex would be the vertex on which the actual evader resides.

Hence, after a sufficiently long period of time the evader is fully localized. The control law in (9), by construction, simply becomes following the negative of the gradient of the square of the geodesic distance to the evader (see first paragraph of Section 4.2). This ensures that the geodesic distance to the evader is decreased at every time-step (formally, the geodesic distance can be considered as a Lyapunov functional candidate the time derivative of which is always negative and zero when the pursuer and the evader are at the same location), hence ensuring the eventual capture of the evader. We summarize this simplified analysis under the following proposition:

Proposition (informal): For a fixed persuer-to-evader assignment, if the evader’s maximum speed is negligible compared to the pursuer’s speed, and if the sensing model for the sensor onboard the pursuer is unbiased, after a sufficiently long period of time the control law in (9) will make the pursuer’s position asymptotically converge to the position of the evader.
